# Peroxidase-like Nanoparticles of Noble Metals Stimulate Increasing Sensitivity of Flavocytochrome *b*_2_-Based L-Lactate Biosensors

**DOI:** 10.3390/bios14110562

**Published:** 2024-11-20

**Authors:** Galina Gayda, Olha Demkiv, Nataliya Stasyuk, Yuriy Boretsky, Mykhailo Gonchar, Marina Nisnevitch

**Affiliations:** 1 Department of Analytical Biotechnology, Institute of Cell Biology National Academy of Sciences of Ukraine, 14/16 Drahomanov Str., 79005 Lviv, Ukraine; demkivo@nas.gov.ua (O.D.); stasukne@nas.gov.ua (N.S.); gonchar@cellbiol.lviv.ua (M.G.); 2 Department of Biochemistry and Hygiene, Ivan Boberskyi Lviv State University of Physical Culture, 11 Kostiushko Str., 79000 Lviv, Ukraine; biolog@ldufk.edu.ua; 3 Department of Chemical Engineering, Ariel University, Kyriat-ha-Mada, Ariel 4070000, Israel

**Keywords:** flavocytochrome *b*_2_, electroactive nanoparticles, nanoperoxidase, amperometric biosensor, L-lactate analysis

## Abstract

We report the development of amperometric biosensors (ABSs) employing flavocytochrome *b*_2_ (Fc*b*_2_) coupled with nanoparticles (NPs) of noble metals on graphite electrode (GE) surfaces. Each NPs/GE configuration was evaluated for its ability to decompose hydrogen peroxide (H_2_O_2_), mimicking peroxidase (PO) activity. The most effective nanoPO (nPO) was selected for developing ABSs targeting L-lactate. Consequently, several Fc*b*_2_/nPO-based ABSs with enhanced sensitivity to L-lactate were developed, demonstrating mediated ET between Fc*b*_2_ and the GE surface. The positive effect of noble metal NPs on Fc*b*_2_-based sensor sensitivity may be explained by the synergy between their dual roles as both PO mimetics and electron transfer mediators. Furthermore, our findings provide preliminary data that may prompt a re-evaluation of the mechanism of L-lactate oxidation in Fc*b*_2_-mediated catalysis. Previously, it was believed that L-lactate oxidation via Fc*b*_2_ catalysis did not produce H_2_O_2_, unlike catalysis via L-lactate oxidase. Our initial research revealed that the inclusion of nPO in Fc*b*_2_-based ABSs significantly increased their sensitivity. Employing other PO mimetics in ABSs for L-lactate yielded similar results, reinforcing our hypothesis that trace amounts of H_2_O_2_ may be generated as a transient intermediate in this reaction. The presence of nPO enhances the L-lactate oxidation rate through H_2_O_2_ utilization, leading to signal amplification and heightened bioelectrode sensitivity. The proposed ABSs have been successfully tested on blood serum and fermented food samples, showing their promise for L-lactate monitoring in medicine and the food industry.

## 1. Introduction

The concentration of L-lactate in real samples is a critical quality indicator for food and beverages [1,2,3,4,5], as well as a biomarker of health in humans and mammals [5,6,7,8,9,10,11,12,13,14,15]. Monitoring L-lactate as a prognostic factor in acute conditions is promising for clinical diagnostics [16,17,18,19,20,21], biomedicine science and biotechnology, medical and veterinary therapy [8,18,22,23,24,25,26,27], and sports medicine for exercise control [28,29,30,31,32,33,34]. Therefore, developing valid, accurate methods for L-lactate assays remains a significant objective.

In recent years, we have seen numerous reports on non-enzymatic sensors based on nanomaterials for L-lactate analysis, but the selectivity of such methods poses challenges, particularly for L-lactate determination in biological fluids [30,35,36,37,38,39,40]. Electrochemical enzymatic L-lactate biosensors, which combine the robustness of electrochemical techniques with the specificity of biological recognition processes, offer significant advantages over conventional analytical techniques in terms of size, cost, sensitivity, selectivity, response speed, and sample pre-treatment, thereby demonstrating broad application prospects. The most effective methods for the L-lactate assay are amperometric biosensors (ABSs) based on enzymes involved in L-lactate metabolism, such as L-lactate oxidase (LOx), L-lactate dehydrogenase (LDH), and L-lactate-cytochrome c oxidoreductase (EC 1.1.2.3, flavocytochrome *b*_2_, Fc*b*_2_) [1,2,3,5,19,41,42,43,44,45,46,47].

The name “flavocytochrome *b*_2_” has more than twenty synonyms, including flavocytochrome *b*, cytochrome *b*_2_, Cy*b*_2_, Cy*b*_2_A, Fc*b*_2_, L-lactate cytochrome c oxidoreductase, L-lactate cytochrome c reductase, L-lactate ferricytochrome C oxidoreductase, cytochrome c oxido reductase, L-lactate dehydrogenase (cytochrome), L-LDH (FMN-dependent), NAD+-independent LDH, iLDH, L-LCO, L-LCR, lldA, LldD, PA4771, and others [48]. The variety of names attributed to this enzyme arises from its discovery across a broad spectrum of organisms. This has resulted in a confusing array of terms and abbreviations that complicate discussions on the topic.

Fc*b*_2_, glycolate oxidase, and several other enzymes belong to the family of FMN-dependent enzymes that oxidize L-2-hydroxy acids to corresponding keto acids. Fc*b*_2_ is a mitochondrial enzyme that catalyzes the oxidative dehydrogenation of L-lactate to pyruvate in yeasts like *Saccharomyces cerevisiae*, *Pichia* (*Hansenula*) *anomala*, and *Ogataea* (*Hansenula*) *polymorpha* [48,49,50,51,52,53,54,55,56]. This reaction is a key step in cellular respiration and energy production, especially under conditions where other more favorable carbon sources than L-lactate might be limited. The physiological electron acceptor of Fc*b*_2_ is cytochrome *c* [49,56,57,58]. Fc*b*_2_ is a homotetrameric enzyme containing a heme *b*_2_ and an FMN group per subunit, located in the N-terminal and C-terminal domains, respectively. During the catalytic cycle, two electrons are transferred from L-lactate to FMN, and then they are transferred one at a time from FMN to heme *b*_2_ [49,50,51,52].

Two primary questions preoccupy the enzymology community concerning Fc*b*_2_: “How does electron transfer (ET) during L-lactate oxidation occur within the enzyme’s subunit?”, and “What is the mechanism of ET both within and across enzyme’s molecules?” [49,50,51,52,56,57,58]. As of now, the detailed mechanism by which Fc*b*_2_ catalyzes L-lactate oxidation remains elusive and subject to debate. This gap in enzymology might be attributed to the absence of Fc*b*_2_ as a commercially available drug. The primary structure of Fc*b*_2_ from the yeast *O. polymorpha* remains unidentified. In addition, there are noted distinctions in the structural and kinetic properties of Fc*b*_2_ from the yeasts *S. cerevisiae* and *P. anomala* [55,56,59,60].

**Figure 1:**
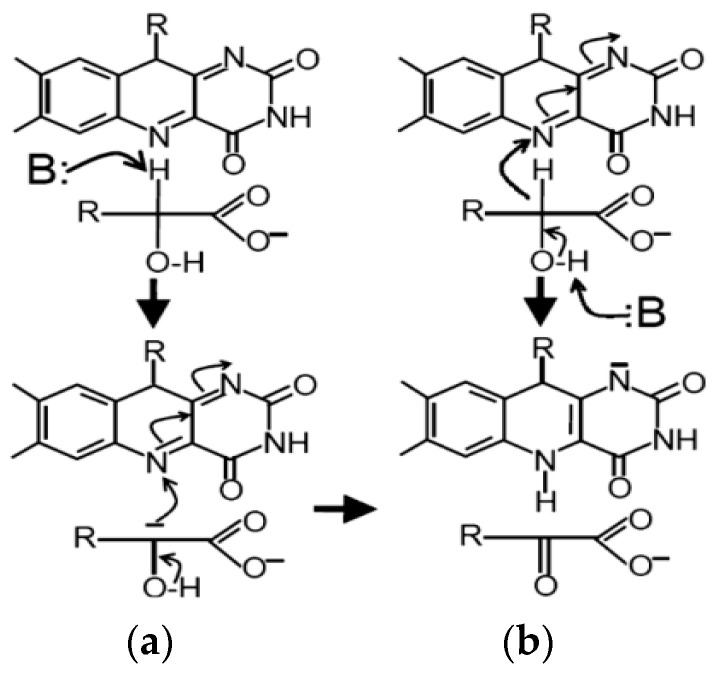
Schemes of L-lactate oxidation under Fc*b*_2_ catalysis according to carbanion (**a**) and hydride (**b**) mechanisms. “B”—a strong base of enzyme active site [50].

Two mechanistic hypotheses for the α-hydroxy acid dehydrogenation reaction, namely the “carbanion” and “hydride transfer” mechanisms, have been formulated and tested to date. Opposing views were discussed in the Lederer and Fitzpatrick groups, respectively [61,62,63,64], with the main points of these debates summarized by Tabacchi [65]. The proposed mechanisms highlight different ways by which L-lactate can be oxidized depending on the catalytic action of Fc*b*_2_ (Figure 1). The “carbanion” mechanism involves the formation of a reactive intermediate at N5 or two consecutive single electron transfer steps, while the “hydride” mechanism involves a direct transfer of electrons as a hydride ion. Florence Lederer, who has extensively investigated yeast Fc*b*_2_ for several decades [58,59,61,65,66], wrote the following: “Flavocytochrome *b*_2_ is a highly unusual dehydrogenase-electron transferase, and one may wonder how its flavin reacts with oxygen” [52]. The last findings by the Lederer group provide compelling evidence supporting the carbanion mechanism of α-hydroxy acid dehydrogenation, particularly focusing on glycolate oxidase [50].

Numerous approaches have been proposed to elucidate the mechanisms of Fc*b*_2_-mediated catalysis. Raman [53] and EPR study [67], gene engineering [3,43,68,69,70,71], protein engineering [72], crystallography [73], site-directed mutagenesis [74,75], MD and QM/MM computations [76], kinetic studies including solvent and pH effects, primary kinetic isotope effects [55,59,60,61,62,74,75], and other techniques were used [77,78,79]. Unfortunately, experimental results often led to ambiguous conclusions.

In our previous work, we reported the development of amperometric biosensors (ABSs) based on Fc*b*_2_ and redox nanoparticles (NPs) with peroxidase-like activity (nPO) [45]. We demonstrated for the first time that ABSs with nPO exhibited significantly enhanced sensitivity compared to ABSs without nPO [45]. The peculiarities of Fc*b*_2_ from various yeasts, including the history of the enzyme’s discovery and research, methods of structural, physicochemical, and catalytic characterization, the development of Fc*b*_2_-based analytical approaches, ways to enhance the sensitivity of Fc*b*_2_-based ABSs, as well as a discussion on possible mechanisms of L-lactate oxidation under enzyme catalysis, were briefly reviewed in this publication [45]. In the current study, we aimed to further investigate the role of peroxidase mimetics (nPO) in Fc*b*_2_/nPO-based ABSs. To this end, we used noble metal NPs with PO-like activities for the construction and characterization of novel L-lactate-sensitive ABSs. We are particularly interested in clarifying whether hydrogen peroxide truly arises as an intermediate during L-lactate oxidation under Fc*b*_2_ catalysis for determining which type of mechanism may be involved in this enzymatic reaction. Our objectives included examining the analytical characteristics of the developed ABSs and testing the most sensitive ABS for L-lactate determination in real food and blood serum samples.

## 2. Materials and Methods

### 2.1. Reagents and Enzyme

Hydrogen tetrachloroaurate(III) trihydrate, chloroplatinic(IV) acid, sodium salt of L-Lactic acid, ascorbic acid, K_3_(Fe(CN)_6_), phenazine methosulfate (PMS), Nafion (5% solution in 90% low-chain aliphatic alcohols), and all other reagents and solvents used in this work were purchased from Sigma-Aldrich (Steinheim, Germany). All chemicals were of analytical grade and were used without additional purification. All solutions were prepared using ultrapure water obtained with the Milli-Q^®^ IQ 7000Water. Purification system (Merck KGaA, Darmstadt, Germany).

Flavocytochrome *b*_2_ (Fc*b*_2_, EC 1.1.2.3) was isolated and purified from the wild strain of thermotolerant methylotrophic yeast *O. polymorpha* 356 up to the specific activity 16 U mg^−1^ and stored as a suspension in 70%-saturated ammonium sulfate at –10 °C as described in detail earlier [4,45].

### 2.2. Apparatus and Statistical Analysis

The evaluation of the ABSs was rigorously conducted using constant-potential amperometry, a methodological approach elaborately detailed in our previous works [45,80]. Each experiment was systematically replicated three times to ensure the reliability and reproducibility of the data. The experimental setup comprised a graphite rod electrode (GE) serving as the working electrode, which has a diameter of 3.05 mm, paired with a platinum wire counter electrode. The reference point for these measurements was established using a Ag/AgCl/KCl (3 M) reference electrode. These components were assembled within a glass electrochemical cell that accommodates a working volume of 20 mL. The analytical performance and parameters of the ABSs (sensitivity, limit of detection (LOD), and other characteristics) were determined based on a statistical methodology published in our previous papers [45,80].

### 2.3. Modification of the Graphite Electrode with Nanoparticles of Noble Metals

#### 2.3.1. Electrodeposition of Nanoparticles

The process of the electrodeposition (ED) of NPs of gold (edAu), platinum (edPt), and a gold/platinum mixture (edAu/Pt) onto the surface of a GE was executed in two primary steps of ED: (1) potentiodynamic and (2) potentiostatic.

The 1st ED phase involved immersing the GE into the corresponding reaction mixture designed for each type of NPs deposition, as detailed in Table 1. The ED was conducted within a specific operating potential range (Table 1) with a scanning rate of 50 mV·s^−1^. As a result, the GE surface was modified with a thin layer of the generated metal edNPs. During the 2nd ED phase, the resulting edNPs/GE was incubated at −1 V within the same reaction mixture for 120 s to achieve a stable thickened film of edNPs on the surface of GE.

#### 2.3.2. Immobilization of Chemically Synthesized Nanoparticles

Other variants of NPs were synthesized through a chemical reduction in their respective acids, HAuCl_4_ and H_2_PtCl_6_, utilizing the reported methods [81]. Following synthesis, the generated NPs of gold (nAu) and platinum (nPt) were separated by centrifugation at 8000× *g* using a Hettich Micro-22R centrifuge, washed three times with distilled water, and subsequently dried at 80 °C for 24 h. The dried nNPs were weighed, resuspended in distilled water to achieve concentrations ranging from 0.1 to 10 mg/mL, and stored at 4 °C for future use.

For the modification of GE, a 5 µL aliquot of nNPs colloid solution (at a concentration of 0.1–10.0 mg/mL) was applied to the surface of the GE. The GEs were either modified (with edAu, edPt, or edAuPt) or unmodified. After air drying, the NPs/GE assembly was coated with 5 μL of a 0.5% neutralized Nafion solution. This film was then dried and the electrode was rinsed with a 50 mM sodium phosphate buffer at pH 8.0 (PB).

As a result, several variants of NPs/GEs were fabricated, including edPt/GE, nPt/GE, nPt/edPt/GE, nAu/edPt/GE, edAuPt/GE, nAu/GE, and nAu/edAuPt/GE. The modified GEs were characterized using cyclic voltammetry (CV) in a potential range from −800 to +800 mV at a scan rate of 50 mV·min^−1^ conducted at room temperature.

The morphological characteristics of the NPs/GEs were scrutinized using scanning electron microscopy (SEM), as described earlier [45,80].

### 2.4. Creation and Characterization of a Biosensor Based on Fcb_2_ and NPs

To develop the bioelectrode Fc*b*_2_/NPs/GE, we used two methods of enzyme immobilization: physical [45] and covalent [82].

Physical adsorption was used for the fabrication of the Fc*b*_2_/GE or Fc*b*_2_/nNPs/GE. For this aim, 10 μL of enzyme solution (with a total activity of 0.3–0.8 units) was applied onto the GE or nNPs/GE, air-dried, and fixed with Nafion film (as described in Section 2.3.2).

For covalent binding, NPs-modified GE was preliminarily activated with glutaraldehyde (GA). To reach this aim, 10 μL of a 1 mM cysteamine solution was applied to the surface of NPs/GE, and after air-drying at room temperature, 10 μL of a 1% GA solution was added. After film formation, the activated NPs/GE were washed several times with 50 mM phosphate buffer, pH 8.0 (PB). Then, 10 μL of Fc*b*_2_ solution (with activity 60–800 units) was applied onto the surface of the GA-activated NPs/GE and air-dried. The fabricated ABSs (Fc*b*_2_/GEs and Fc*b*_2_/NPs/GEs) were rinsed with PB; their electrochemical properties were evaluated following the methodology that was previously described [45].

### 2.5. Evaluation of the Main Operating Characteristics of the Developed Biosensors

The Fc*b*_2_/GE and Fc*b*_2_/NPs/GE systems underwent detailed characterization, including evaluation of the main operating characteristics according to the main parameters: a value of the highest current response when saturated with the L-lactate (I_max_); a value of the apparent Michaelis–Menten constant (K_M_^app^); and the limit of detection (LOD), linearity, and sensitivity to L-lactate.

Values for I_max_, K_M_^app^, and the correlation coefficient of linear regression (R) were calculated automatically and placed in the wide-range calibration curve field. Sensitivity characterizes the specific activity of the ABS; its value was calculated as the ratio of the slope “B” value (from the linear regression graph) to the square of the active GE surface (7.3 mm^2^) and expressed in standard SI units, A·M^−1^·m^−2^. The LOD was determined as a ratio of the triplicated standard deviation value of the blank to the slope “B”.

### 2.6. Biosensor L-Lactate Analysis in Real Samples

The ABS demonstrating the highest sensitivity was employed for the analysis of L-lactate in various real samples, including blood serum, commercial yogurt, and cucumber brine. To ensure reliability, each analysis was conducted in triplicate across two different dilutions for each sample type.

To prepare a blood serum sample, venous blood was collected from a cohort of three healthy female volunteers, all aged 35–40 years. The collected blood was incubated at 8 °C for 30 min, followed by centrifugation at 3000 rpm for 5 min using a Micro 22R centrifuge (Hettich Zentrifugen, Tuttlingen, Germany). A clear supernatant (serum) was taken and stored at −20 °C until analysis.

Food samples, including yogurt and cucumber brine, were prepared in a manner similar to serum samples by subjecting them to centrifugation under identical conditions.

To mitigate potential interference from other components in the samples, a graphical calibration method incorporating the standard addition test (SAT) was utilized. This method, along with the algorithm for calculating the analyte concentration (C) using the formula C = A·N/B (where A and B are linear regression parameters and N is the dilution coefficient), has been detailed in our prior publications [45,80,82]. The calibration graphs provided the necessary values for these calculations.

For comparative purposes, an optical enzymatic–chemical method based on Fc*b*_2_ was employed as a reference. The methodology, extensively described in our recent study [45], involved measuring the optical density of the resultant solution at 680 nm using an SHIMADZU UV-1650 PC spectrophotometer (Kyoto, Japan) equipped with the “UV Probe 2.20” software against a blank sample.

## 3. Results

### 3.1. Production and Characterization of Electroactive Nanomaterials

**Figure 2:**
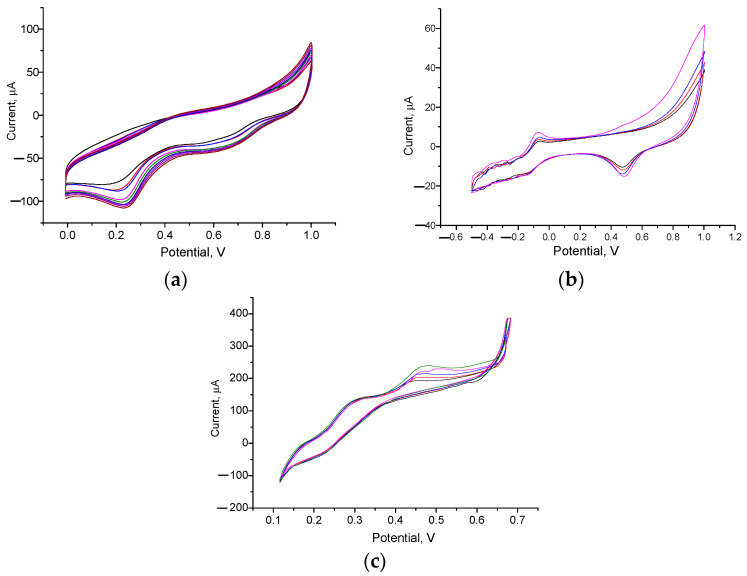
CV profiles of the electrodeposition process for obtaining edPt (**a**), edAu (**b**), and edAu/Pt (**c**) on the GE surface. The black line represents the 1st cycle, while the red, blue, and pink lines correspond to the 2nd, 3rd, and 4th cycles, respectively, and so forth.

In the realm of biosensing, the integration of synthetic NPs within the biorecognition layer as electronic mediators has emerged as a transformative approach to significantly boost the sensitivity of ABSs [45,80,82]. This enhancement is primarily attributed to the acceleration of ET rates facilitated by these NPs. Our investigations have focused on examining various GEs that have been adeptly modified with NPs of noble metals, utilizing CV as the primary method of characterization. These modifications were systematically applied to the surfaces of GEs, as outlined in Section 2.3. The corresponding CV profiles of several edNPs/GEs are shown in Figure 2.

**Figure 3:**
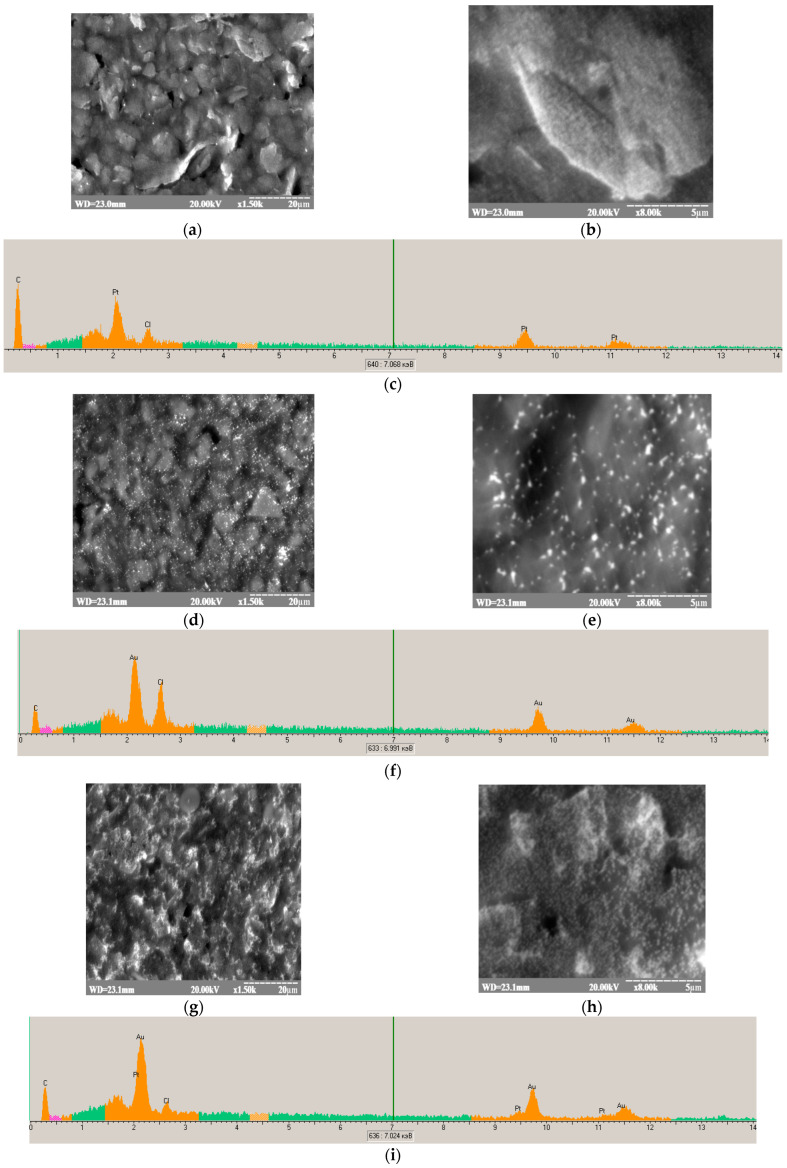
SEM imaging and X-ray spectral microanalysis of edPt (**a**–**c**), edAu (**d**–**f**), and ed Au/Pt (**g**–**i**) on the surface of the GE. The characteristic emission peaks, corresponding to specific chemical elements, are highlighted in orange (**c**,**f**,**i**).

A detailed examination of the electrode’s morphology is essential for optimizing the design and fabrication of highly sensitive and efficient ABSs, leveraging the unique properties of NPs to enhance biorecognition and ET processes. The morphology of the modified GEs was meticulously analyzed using SEM, providing valuable information on the size, distribution, and shape of the tested materials. Figure 3 presents the results of the morphological characterization of edPt (Figure 3a,b), edAu (Figure 3d,e), and edAu/Pt (Figure 3g,h).

X-ray microanalysis (XRM) is commonly used to confirm the elemental composition of nanomaterials, including the presence of Pt and Au nanoparticles. The XRM images (Figure 3c,f,i) confirmed the presence of all components in the tested nanomaterials. It is known that the primary X-ray emission peaks indicating the formation of Pt⁰ are approximately 9.44 keV (Pt Lα), 11.07 keV (Pt Lβ), and 2.05 keV (Pt Mα). These peaks, shown in Figure 3c,i, confirm the presence of Pt in the synthesized nanomaterial and differentiate it from other elements. Au^0^ formation has been proved by the characteristic emission peaks at 2.1, 9.7, and 11.6 keV corresponding to the AuK_α_, AuK_α_, and AuK_β_ transitions, respectively (Figure 3f,i).

The nanoscale dimensions, which influence surface area and the exposure of active sites, directly affect the catalytic efficiency of these NPs [35,37,83,84]. Smaller NZs, with their larger surface-area-to-volume ratio, provide more active surface sites, which can enhance interactions with substrates and improve catalytic efficiency. Additionally, the shape and overall morphology of the NPs also play an essential role as they influence how the nanoparticles interact with their environment and with substrates, further contributing to their catalytic performance. The high surface roughness of electrodeposited NPs is likely one of the factors contributing to their elevated catalytic activity. In addition to their size, the composition of NPs is indeed a crucial factor in determining their catalytic properties [85,86,87]. Chemical modifications, such as doping or alloying, can significantly alter the electronic structure of the NPs, influencing their reactivity and stability. These compositional changes impact the availability of active sites on the surface, thereby affecting their catalytic efficiency; the combination of surface structure and composition is pivotal in determining the overall catalytic efficiency of NPs, including electrodeposited ones [88,89].

The SEM and DLS results, which were presented in our previous paper, indicate the formation of spherical particles of nPt and nAu with the average diameter <100 nm [81]. According to Figure 3, the size distributions of the edNPs were found to range between 20 nm and 200 nm. This variance in size plays a critical role in defining the surface area available for ET reactions and the overall electroactivity of the electrode. Smaller NPs can provide a higher surface-area-to-volume ratio, potentially leading to enhanced ET rates and the improved sensitivity of the ABS. Conversely, larger NPs may influence the system differently, potentially by stabilizing the GE surface or affecting the distribution of electroactive sites.

**Figure 4:**
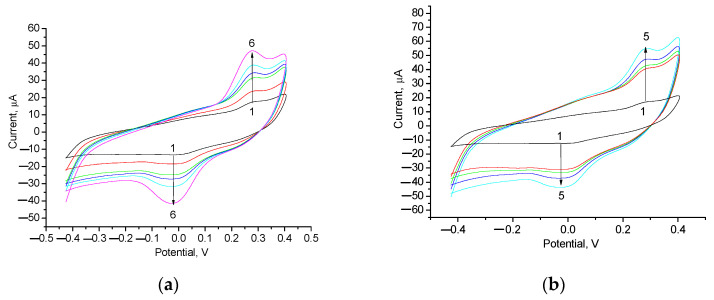
CV profiles of the modified electrodes with architectures nPt/GE (**a**) and nAu/edAuPt/GE (**b**) as outputs on increasing H_2_O_2_ concentration (mM): 1–0 (black); 2–0.5 (red); 3–1 (green); 4–5 (blue); 5–9 (cyan); 6–16 (violet). Conditions: –0.4 V to 0.4 V vs. Ag/AgCl; scan rate 7 mV/s in 50 mM phosphate buffer, pH 8.0.

nAu and nPt exhibit catalase-like activity [81] and PO-like activity in solution. Electrochemical experiments demonstrated that all the developed NP-modified GEs had the ability to decompose H_2_O_2_, exhibiting various levels of activity. The highest current signals in response to the addition of H_2_O_2_ were demonstrated by the nPt/GE and nAu/edAuPt/GE electrodes. Their CV profiles are shown in Figure 4. According to the CV results for the best electrode nAu/edAuPt/GE (Figure 4b), the peak of oxidation, as an output upon H_2_O_2_ addition, appeared in the range of 150–400 mV. Thus, the optimal working potential for studying all the NPs/GEs was selected as +200 mV.

### 3.2. Development and Characterization of Biosensor

In the current work, various types of ABSs with the Fc*b*_2_/NPs/GE architecture have been developed. Fc*b*_2_ is a tetrameric molecule with eight folded domains, and it needs to be effectively immobilized on the surface of the GE. To enhance the efficiency of ET and to ensure the MET between the enzyme and the electrode, the surface of the GE was modified by electrochemically active NPs with PO-like activity.

The fabricated ABSs incorporated various methods for NP modification on the GEs. These methods included electrodeposition followed by the formation of edAu, edPt, and edAuPt films, physical immobilization of chemically synthesized NPs such as nAu and nPt, as well as a combination of electrodeposition and adsorption procedures. The enzyme on the surface of the NPs/GE was immobilized by physical adsorption, followed by fixation with a Nafion film, or by covalent immobilization on the GA-activated NPs/GE surface.

**Figure 5:**
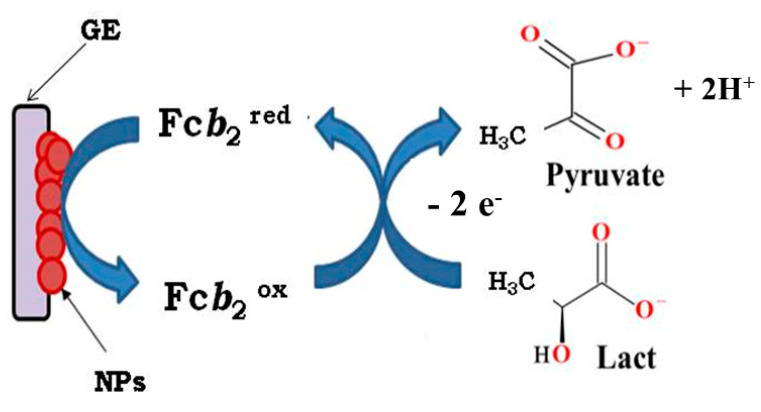
The schematic diagram of electron transfer in the Fc*b*_2_/NPs/ABS in the presence of L-lactate as a substrate [45].

The scheme illustrating the operation of Fc*b*_2_/NPs/ABS for L-lactate analysis was proposed by us earlier [45] and is presented in Figure 5.

**Figure 6:**
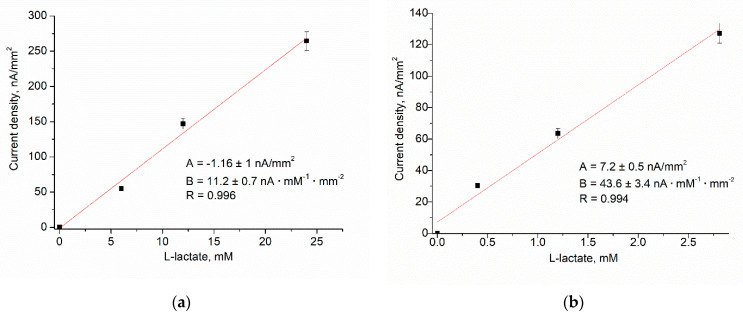
Calibration curves illustrating the current dependence on the L-lactate concentration for the ABS with the Fc*b*_2_/GE architecture in the absence (**a**) and in the presence of 1 mM PMS (**b**). Conditions: +200 mV, 0.3 units Fc*b*_2_.

While Fc*b*_2_ is capable of DET on the electrode surface [45,83,84,85], the efficiency of this process is relatively low (Figure 6a). Fc*b*_2_ is a unique enzyme known for its absolute selectivity towards L-lactate, yet it operates in the presence of various electron acceptors, including 5-methylphenazinium methyl sulfate (PMS). Consequently, to enhance the electrochemical communication between the immobilized Fc*b*_2_ and the GE surface, we used a low-molecular-weight free-diffusing redox mediator PMS (Figure 6b). Due to its relatively stable nature, lack of reactivity with L-lactate, and ability to undergo reversible redox reactions, PMS is commonly used in enzymatic assays, ABSs, and enzymatic biofuel cells.

We have demonstrated in our previous work that the increased ET in Fc*b*_2_-based ABS may be achieved due to MET by using immobilized electroactive NPs of special shape and size [45]. Since Fc*b*_2_ is a large multi-domain enzyme, for its immobilization, a matrix with small-size particles is needed. SEM images (Figure 3) proved that the obtained NPs of noble metals are, namely, such materials.

We studied the effect of the PO-like NPs (nPO) on the analytical properties of the developed ABSs, which differed in terms of their sensing films, particularly in the composition of the NPs and the amount of immobilized Fc*b*_2_. The current responses of different ABSs to the added substrate (L-lactate) were compared using CV and chronoamperometry; however, detailed descriptions for all ABSs are omitted here. The calibration was performed by the stepwise addition of L-lactate solution. Following the chronoamperograms, calibration curves for L-lactate determination in wide and linear ranges were plotted.

**Figure 7:**
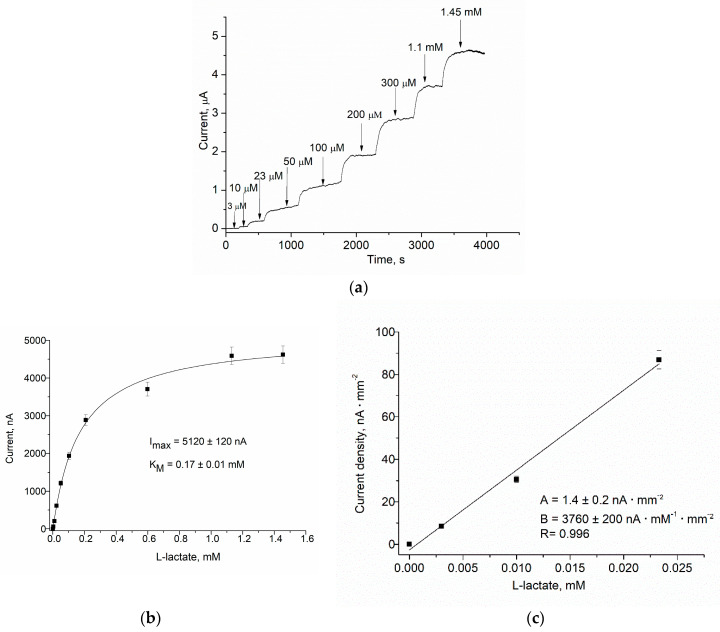

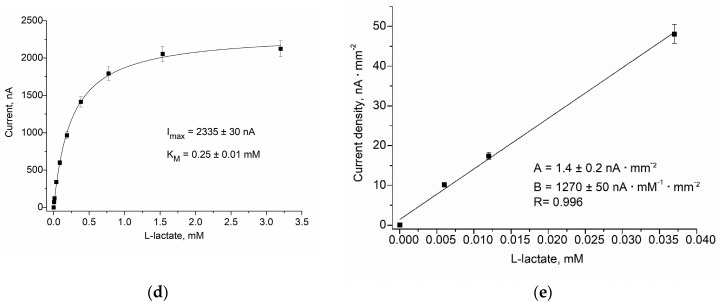
Characterization of the ABSs with the architecture Fc*b*_2_/nAu/edAuPt/GE in the presence of 1 mM PMS: (**a**)—chronoamperogramm, (**b**–**e**)—calibration graphs in wide (**b**,**d**) and linear (**c**,**e**) ranges, respectively. The ABSs contain 0.8 units (**a**–**c**) and 0.3 units (**d**,**e**) of Fc*b*_2_ in their biorecognition layers. Conditions: working potential is +200 mV, 1 mM PMS.

As a result, the Fc*b*_2_/nAu/edAuPt/GE, Fc*b*_2_/nPt/GE, and other proposed NPs containing ABSs have demonstrated superior operational characteristics (Figure 7, Figures S1 and S2) compared to control bioelectrode Fc*b*_2_/GE (Figure 6). The amperometric characteristics of the ABS with the highest sensitivities (Fc*b*_2_/nAu/edAuPt/GE) and other ABSs are summarized in Table 2.

According to the data presented in Table 2, the highest sensitivity (3760 A·M^−1^·m^−2^) was achieved with 0.8 units of Fc*b*_2_ on the nAu/edAuPt/GE surface. The ABS with the same composition but a 2.7-fold lower enzyme amount (0.3 units) exhibited a 3-fold lower sensitivity (1270 A·M^−1^·m^−2^). The last value of sensitivity is significantly higher compared to the ABSs with an identical amount of enzyme, being 5 times higher than that of the Fc*b*_2_/edAuPt/GE and 30 times higher than the control Fc*b*_2_/GE, tested under identical conditions, in the presence of PMS.

The main analytical properties of the most effective Fc*b*_2_-NPs-based ABS developed in the current work indeed surpass those previously reported [45], particularly in terms of sensitivity and limit of detection (LOD). Specifically, the sensitivity of the ABS with the nAu/edAuPt/GE is 2.6 times higher, and the LOD is 5 times lower than that of the most effective ABS with architecture Fc*b*_2/_PtZn/GE [45].

In our recent works, we have demonstrated the significance of optimizing the enzyme amount and the ratio of enzyme/nPO on the surface of the electrode [45,80,86]. This conclusion will prove valuable for researchers utilizing large multi-subunit enzymes in the development of ABSs. However, in this research, enhancing ABS sensitivity due to the selection of the optimal conditions is not our primary aim.

The selectivity of the ABSs toward the target analyte is crucial, especially for analyzing real samples. Yeast Fc*b*_2_ is known to exhibit high selectivity toward L-lactate [3,90]. We demonstrated earlier the high selectivity of Fc*b*_2_-based ABSs to L-lactate by testing a variety of organic substances [45]. It is worth mentioning that the NPs/GE described here did not react with all the tested analytes. Notably, co-immobilization of Fc*b*_2_ with NPs on the GE surface led to enhanced storage stability of the ABSs compared to those without NPs [3,45]. To study the selectivity of the proposed Fc*b*_2_/nAu/edAuPt/GE, this ABS has been tested for its ability to respond to several individual compounds which are usually contained in biological liquids and food products, e.g., organic acids and glucose. As can be seen from Figure S3, there are no significant current signals for analytes other than L-lactate. These results confirm the high selectivity of the proposed ABS for L-lactate detection.

### 3.3. L-Lactate Determination in the Real Samples with the Fcb_2_/nAu/pAuPt/GE

Lactic acid is an organic acid found in the human body and in fermented foods. First of all, rapid analysis of L-lactate is necessary for traumatology, critical and emergency medicine, anesthesiology, intensive care and surgery, endocrinology, maternity hospitals, and perinatal centers, as well as sports medicine. The L-lactate level is a prognostic factor for death in the emergency department, sepsis, shock, and burns [1,2,41,42]. Additionally, L-lactate is commonly found in dairy products and plays a significant role in various technological processes. It serves as a primary component affecting the taste, pH, and emulsification stability of food products.

**Figure 8:**
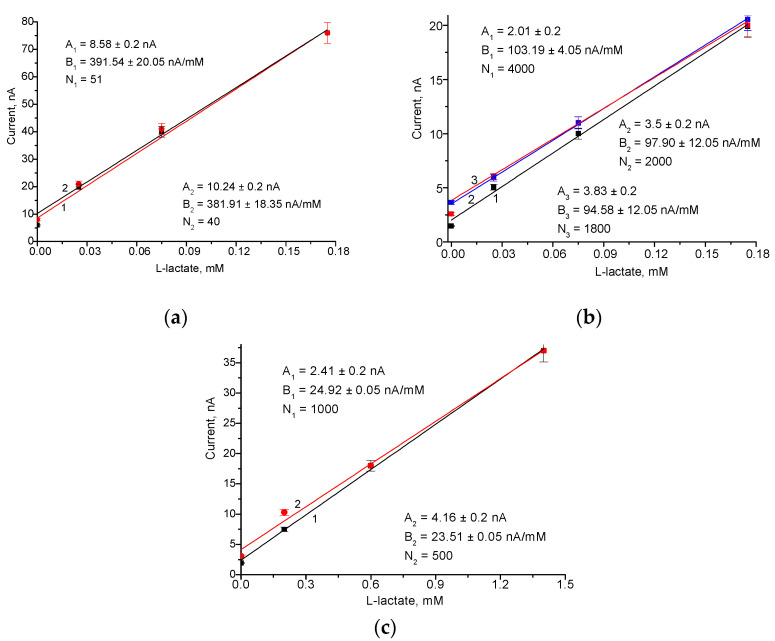
The quantification of L-lactate using Fc*b*_2_/nAu/edAuPt/GE in the samples of blood serum (**a**) and fermented foods, specifically yogurt (**b**) and cucumber brine (**c**).

To assess the practical application of the developed ABS in analyzing real samples, Fc*b*_2_/nAu/edAuPt/GE was utilized to detect L-lactate in a pool of blood serum from healthy donors as well as in various beverage foods, including in yogurt (Halychyna LLC, Lviv, Ukraine) and cucumber brine (homemade). Real samples at various dilutions were tested using the graphical method known as the standard addition test (SAT). The results of this study are presented in Figure 8.

The graphical SAT (see Section 2.6) is a quantitative analysis method often used in analytical chemistry, where a known standard is added directly to aliquots of the sample being analyzed. The SAT is applied in cases where sample components may contribute to the analytical signal, making routine calibration methods ineffective. The L-lactate content in the original sample was calculated using the equation C = A·N/B, where A and B are parameters of the linear regression of the corresponding calibration graph, and N is the dilution factor of the tested sample.

The average concentrations of L-lactate in the tested samples, as estimated using the ABS, are summarized in Table 3. These values are compared with data obtained through a reference enzymatic–chemical method for validation and performance benchmarking. It is evident that the values of L-lactate content determined by both methods are in good agreement with each other and with the published data (Table 3).

As can be seen from Table 3, the obtained results confirm the adequate accuracy of the ABS approaches for L-lactate analysis (with differences of less than 5.0%), indicating their potential for medical diagnostics and food quality control.

Correlation between the results of L-lactate content determination in samples of serum, yogurt, and cucumber brine using the biosensor and reference methods is presented in Figure S4, demonstrating the applicability of the developed ABS for the L-lactate assay in real samples of biological fluids.

## 4. Discussion

In the current and previous works, we emphasized the superior features of Fc*b*_2_-based ABSs over traditional devices for L-lactate analysis, which primarily rely on LOx or LDH as biorecognition elements [3,4,45,95]. The key benefits of employing Fc*b*_2_ from *O. polymorpha* in analytical methods include cost-effectiveness and procedural simplicity. This is attributed to the use of a single enzyme, eliminating the need for cofactors required by LDH-based methods and simplifying the process compared to LOx-based approaches. Another significant advantage of the proposed Fc*b*_2_/NPs/ABSs is their straightforward sensing layer architecture and the capability to function at low potentials without dependence on oxygen. This is a notable improvement over bacterial LOx, enhancing the system’s applicability and efficiency. Finally, the primary advantage of Fc*b*_2_ isolated from the thermotolerant methylotrophic yeast *O. polymorpha* is its stability at elevated temperatures and in daylight, which surpasses that of Fc*b*_2_ derived from other yeasts, particularly *S. cerevisiae*, whose enzyme is highly unstable in the presence of air.

Moreover, the use of electroactive NPs as both ET mediators and immobilization carriers for the enzyme in ABSs presents economic, stable, and easy-to-synthesize alternatives to the complex multi-layer constructions often encountered in the ABSs [1,2,5,16,33,40,41,42,46].

The possibility of DET from the reduced form of yeast Fc*b*_2_ to the surface of the electrode (GE) has been demonstrated by us earlier [3,45,95] and in this study. Electroactive metallic NPs with PO-like activity (nPO) immobilized on the GE stimulate the increasing sensitivity of the Fc*b*_2_-based ABS due to enhanced mediated electron transfer (MET) or other reasons. Most oxidase-based ABSs can operate effectively only if they contain natural or artificial PO, coupled with the oxidase. It is understandable that these PO are capable of decomposing H_2_O_2_, a common product of oxidase function.

Fc*b*_2_ is indeed not an oxidase. Therefore, it was assumed that it does not generate H_2_O_2_ as a by-product of catalytic L-lactate oxidation. However, we have demonstrated for the first time recently [45] and reaffirmed this result in the current study that ABS with architecture Fc*b*_2_/nPO/GEs possess significantly enhanced sensitivity in comparison to those with Fc*b*_2_/GE alone.

The same conclusion was made for ABSs, which contain laccase and nPO [82]. To date, the exact pathway mechanisms in laccase catalytic activities are still unknown too. The issue regarding the possible mechanisms of laccase-mediated catalysis we have reviewed in our previous work [82]. Due to high-throughput physical methods used in investigating the ET mechanism, including crystallographic studies utilizing high-intensity X-ray synchrotron beam radiation, the hypothesis of the formation of a ‘peroxide’ intermediate (PI) in laccase-mediated catalysis has been formulated and validated [82].

Traditionally, it is understood that both Fc*b*_2_ and laccase do not require H_2_O_2_ as a co-substrate or an additional cofactor for enzymatic reactions, nor do they produce this compound during oxidation of their substrates. However, this postulate may be disproven, when the exact mechanisms of O_2_ reduction mediated with yeast Fc*b*_2_ and fungal laccase will be studied in detail.

The chemical basis of oxygen reactivity in flavoenzymes continues to be a challenging topic in modern flavoenzymology [49,52,56,63,75,77,96,97]. Studies have shown that both the separately engineered flavodehydrogenase domain and the tetrameric holoenzyme can produce superoxide anions during a very slow reaction when oxygen acts as the sole electron acceptor [52,96,97].

In our current study, we demonstrated once again that incorporating electroactive nPO into the sensing layer of Fc*b*_2_-based ABSs enhances the analytical performance of these biosensors. We hypothesize that the formation of a short-lived PI during Fc*b*_2_-catalyzed dehydrogenation of L-lactate cannot be excluded. During this process, the carbanion formed at the α-carbon of L-lactate can reduce molecular oxygen (in the absence of cytochrome c as the natural electron acceptor), leading to the formation of H_2_O_2_. Several sources in the literature report results similar to ours [52,71]. So, if a PI indeed forms during this reaction, then the role of nPO in increasing the efficiency of DET and MET becomes clearer: the nPO decomposes H_2_O_2_, thereby enhancing the catalytic efficiency and overall sensitivity of the ABS.

We believe that our findings, obtained through a simple electrochemical approach, indirectly support the concept of peroxide intermediate generation during the catalytic process of Fc*b*_2_. Further investigations can help to support this hypothesis. To identify and characterize the short-lived peroxide intermediate, advanced techniques such as spectroscopy (e.g., EPR or UV-Vis), mass spectrometry, protein engineering, and crystallography, as well as SEM in the redox competition mode, should be utilized [43,47,50,53,59,72,98]. Computational modeling, including quantum mechanical or molecular dynamics simulations, can also be beneficial for predicting the energy states and lifetimes of these intermediates, offering deeper insights into the reaction pathways involved [76,77,78,79].

Since the enzyme from the yeast *O. polymorpha* is poorly investigated, it is important to fundamentally study the Fc*b*_2_ structure and its catalytic mechanism. This knowledge will provide a better understanding of how nature controls the process of O_2_ reduction to H_2_O and may be of practical importance for biomedical applications and industrial processes.

## 5. Conclusions

In the current work, we used noble metal nanoparticles with pseudo-peroxidase activities (nPO) for the construction and characterization of novel L-lactate-sensitive amperometric biosensors with architecture Fc*b*_2_/nPO/GE. The most efficient Fc*b*_2_/nAu/edAuPt-based biosensor with a remarkable sensitivity of 3760 A·M^−1^·m^−2^ was adept at analyzing L-lactate in samples of blood serum, yogurt, and cucumber brine. A high correlation was observed between the L-lactate levels determined by this biosensor and those obtained using reference methods. Our results suggest that the Fc*b*_2_/nPO-based biosensors are very promising for applications in clinical diagnostics and food control laboratories.

Moreover, the superior sensitivity of Fc*b*_2_/nPO-containing biosensors reaffirms the importance of these nanomediators and provides preliminary data that may prompt a re-evaluation of the catalytic oxidation mechanism of L-lactate by Fc*b*_2_.

We were particularly interested in clarifying whether hydrogen peroxide truly arises as an intermediate during L-lactate oxidation under Fc*b*_2_ catalysis for determining which type of mechanism may be involved in this enzymatic reaction. We hypothesize the appearance of a short-lived peroxide intermediate in the carbanion mechanism of α-hydroxy acid dehydrogenation under Fc*b*_2_ catalysis. We also cannot rule out an alternative explanation for the positive effect of peroxidase-like mimetics on Fc*b*_2_-based sensor outputs, namely their electron transfer activity as mediators. Perhaps the synergy between the dual activities of nPO has a crucial effect. Though further investigations using high-throughput techniques are necessary to fully address this scientific problem, we hope that our insights contribute, albeit modestly, to elucidating the mechanism of Fc*b*_2_ catalytic activity.

## Figures and Tables

**Table 1:** Conditions for potentiodynamic electrodeposition of NPs on the GE.

NPs	Composition of Reaction Mixture	Potential Range, V	Cycles Number
edAu	10 mM HAuCl_4_ in 2.5 M NH_4_Cl	from −0.5 to 1	4
edPt	10 mM H_2_PtCl_6_ in 2.5 M NH_4_Cl	from 0 to 1	9
edAu/Pt	30 mM HAuCl_4_ and 30 mM H_2_PtCl_6_ in 2.5 M NH_4_Cl	from 0.1 to 0.7	8

**Table 2:** The analytical characteristics of the ABSs with the Fc*b*_2_/NPs/GE.

ABS	Biorecognition Layer	PMS	Activity, Units		Sensitivity, A·M^−1^·m^−2^	LOD, µM
			Fc*b*_2_	nPO		
1	Fc*b*_2_	−	0.3	0	11	100
2		+			44	50
3	Fc*b*_2_/nAu	+	0.8	0.45 × 10^−3^	52	50
4	Fc*b*_2_/nPt		0.3	1 × 10^−3^	1436	5
5				0.13 × 10^−3^	165	20
6	Fc*b*_2_/edPt			not determined	342	10
7	Fc*b*_2_/edAuPt		0.8		266	0.1
8	Fc*b*_2_/nAu/edPt			0.45 × 10^−3^	647	10
9	Fc*b*_2_/nAu/ edAuPt				3760	0.6
10			0.4		1270	1.1
11		−			337	1
12			0.8		22	100

**Table 3:** L-lactate concentration in the tested samples analyzed using two methods.

	Method	Fc*b*_2_/nAu/edAuPt/GE	Reference Method	Published	Fc*b*_2_/nAu/edAuPt/GE	Reference Method	Published
Sample		L-Lactate, mM	^1^ CV, %	L-Lactate, mM	L-Lactate, mM	^1^ CV, %	L-Lactate, mM
Blood serum		1.1 ± 0.05	4.5	1.5 ± 0.05	3.3	0.5–20	[90]
Yogurt		74.3 ± 3.6	4.8	72 ± 2.5	3.5	65–120	[91]
Cucumber brine		93 ± 4.0	4.3	95 ± 4.6	4.8	50–140	[92,93,94]

^1^ CV—variation coefficient.

## Data Availability

The data are included within the present article.

## Supplementary Materials

The following supporting information can be downloaded at https://www.mdpi.com/article/10.3390/bios14110562/s1. Figure S1. The chronoamperogramm (**a**) and calibration graphs (**b**,**c**) of current dependence on L-lactate concentration for ABS with Fc*b*_2_/edAuPt/GE architecture. Conditions: +200 mV, 0.8 units Fc*b*_2_, in the presence of 1 mM PMS. Figure S2. Calibration graphs of current dependence on L-lactate concentration for the ABSs with the Fc*b*_2_/nAu/edPt/GE (**a**,**b**) and Fc*b*_2_/nAu/GE (**c**,**d**) architectures in both the wide (**a**,**c**) and linear (**b**,**d**) ranges. Conditions: +200 mV, 0.8 units Fc*b*_2_, in the presence of 1 mM PMS. Figure S3. The selectivity test for Fc*b*_2_/nAu/edAuPt/GE: relative current outputs (in %) on the compounds that were added up to 2 mM. Figure S4. Correlation between the results of L-lactate content determination (mM) in samples of serum (1), yogurt (2), and cucumber brine (3) using the Fc*b*_2_-based biosensor and reference methods.
