# Supplementary material for: Peroxidase-like Nanoparticles of Noble Metals Stimulate Increasing Sensitivity of Flavocytochrome b2-Based L-Lactate Biosensors
Source: Biosensors (Basel). 2024 Nov 20;14(11):562. doi: 10.3390/bios14110562 (PMC11591947; doi:10.3390/bios14110562)
Supplement: Supplementary file 1 [file biosensors-14-00562-s001.zip › biosensors-3199714-supplementary.pdf]

Supplementary Information

Peroxidase-Like Nanoparticles of Noble Metals Stimulate Increasing Sensitivity of Flavocytochrome *b*$_2$-Based L-Lactate Biosensors

Galina Gayda [1,*], Olha Demkiv [1], Nataliya Stasyuk [1], Yuriy Boretsky [2], Mykhailo Gonchar [1] and Marina Nisnevitch [3,*]

[1] Department of Analytical Biotechnology, Institute of Cell Biology National Academy of Sciences of Ukraine, 14/16, Drahomanov Str., 79005 Lviv, Ukraine. galina.gayda@nas.gov.ua (G.G.); demkivo@nas.gov.ua (O.D.) stasukne@nas.gov.ua (N.S.); gonchar@cellbiol.lviv.ua (M.G.)

[2] Ivan Boberskyi Lviv State University of Physical Culture, 11 Kostiushko St., Lviv 79000, Ukraine. biolog@ldufk.edu.ua

[3] Department of Chemical Engineering, Ariel University, Kyriat-ha-Mada, Ariel 4070000, Israel. marinan@ariel.ac.il

* Correspondence: galina.gayda@nas.gov.ua, galina.gayda@gmail.com (G.G.); marinan@ariel.ac.il (M.N.)

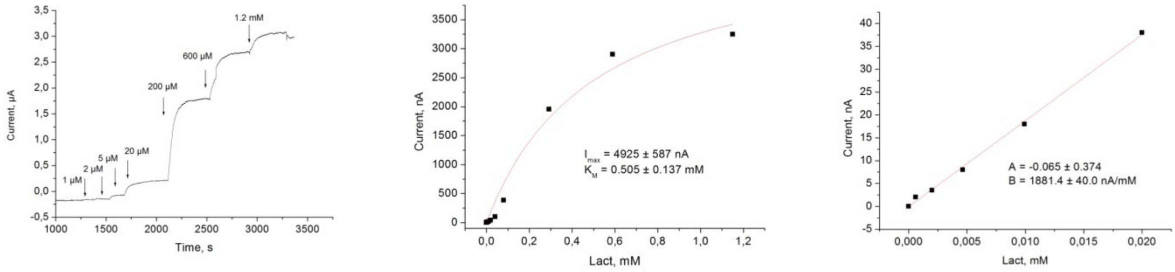

(**a**) (**b**) (**c**)

Figure S1. The chronoamperogramm (**a**) and calibration graphs (**b**-**c**) of the current dependence on L-lactate concentration (labeled as Lact in the figure) for ABS with an Fc*b*$_2$/edAuPt/GE architecture. Conditions: +200 mV, 0.8 units Fc*b*$_2$, in the presence of 1 mM PMS.

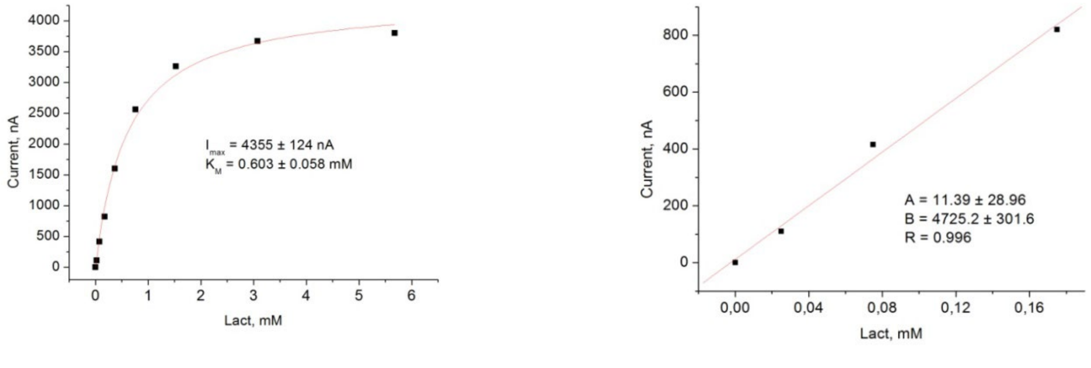

(**a**) (**b**)

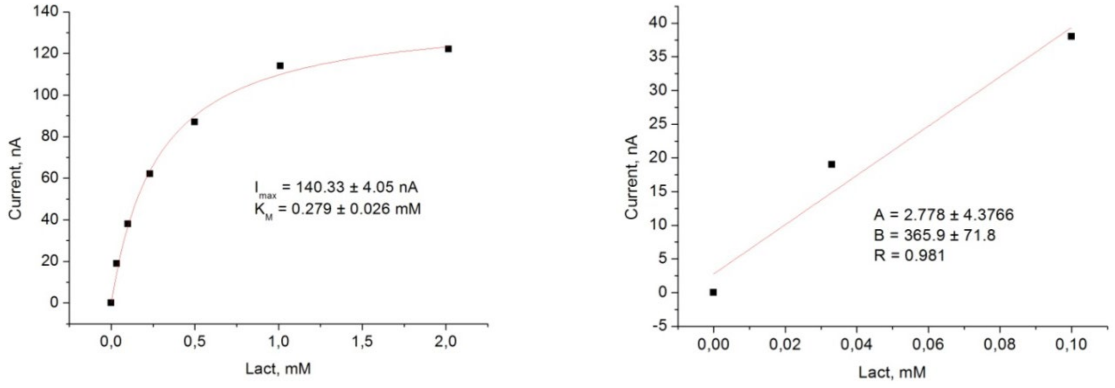

(**c**) (**d**)

Figure S2. Calibration graphs of the current dependence on L-lactate concentration (labeled as Lact in the figure) for the ABSs with the Fc*b*2/nAu/edPt/GE (**a**-**b**) and Fc*b*2/nAu/GE (**c**-**d**) architectures in both the wide (**a**, **c**) and linear (**b**, **d**) ranges. Conditions: +200 mV, 0.8 units Fc*b*2, in the presence of 1 mM PMS.

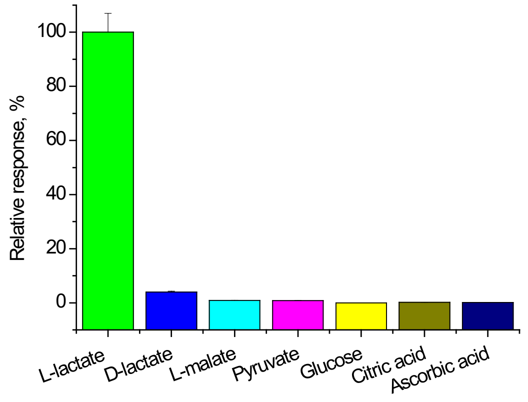

Figure S3. The selectivity test for Fc*b*2/nAu/edAuPt/GE: relative current outputs (in %) on the compounds that were added up to 2 mM.

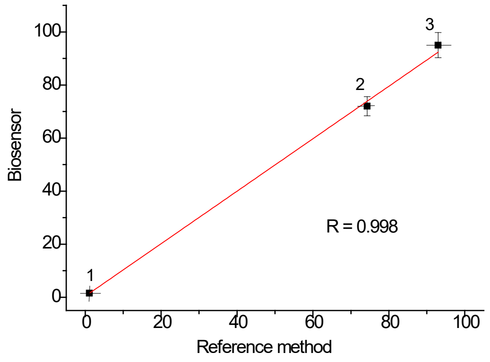

Figure S4. Correlation of L-lactate content (mM) in serum (1), yogurt (2), and cucumber brine (3) samples as determined by the Fc*b*2-based biosensor and reference methods.
